# Supplementary material for: A highly sensitive protein-RNA cross-linking mass spectrometry workflow with enhanced structural modeling potential
Source: Nucleic Acids Res. 2025 Jun 18;53(11):gkaf523. doi: 10.1093/nar/gkaf523 (PMC12203917; doi:10.1093/nar/gkaf523)
Supplement: gkaf523_Supplemental_Files [file gkaf523_supplemental_files.zip › NAR-01789-Met-G_2024_R1_SI.docx]

## **Supplementary Data**

## ***Supplementary Tables***

Supplementary Table 1: Result summary of all experiments discussed in this work. Provided as a separate table in Microsoft Excel xlsx format.

Supplementary Table 2: Putative RNA-derived peptide adduct types identified in open modification search not validated by xQuest search.

| **Mass addition to peptide (Da)** | **Putative explanation** | **Light form detected in open search** | **Heavy form detected in open search** | **Previous evidence in literature** | **Rationale for exclusion from further xQuest searches** |
| --- | --- | --- | --- | --- | --- |
| 151 | Guanine nucleobase only (loss of sugar and phosphate) | Yes | N/A | Panhale 2019(1) | Nucleotides in FOX1-UGCAUGU, experiments are labelled with ^13^C ribose. A nucleobase without ribose exhibits no shift. |
| 712/727  (Light/ Heavy) | Former Uxx trinucleotide, light, with loss of 2 bases; residual backbones from 2 further nucleotides (including labelled ribose) remain | Yes | No | Kramer 2011(2), Panhale 2019(1) | Challenging to encode in parameters where ribose is labelled; similar information content to a mononucleotide without residual backbones |
| 711/726  (Light/ Heavy) | Former Cxx trinucleotide, light, with loss of 2 bases; residual backbones from 2 further nucleotides (including labelled ribose) remain | Yes | No | Kramer 2011(2), Panhale 2019(1) | Challenging to encode in parameters where ribose is labelled; similar information content to a mononucleotide without residual backbones |

Several other nucleotide-derived adducts are putatively identified in the open modification search of CLIR-MS data generated from the FOX1-FBE complex. However, these were not subjected to further validation with an xQuest search, nor are they routinely encoded as default loss products for future xQuest searches, owing to their practical incompatibility with the CLIR-MS approach, and the atoms carrying the isotope label in each nucleotide.

Free nucleobases other than guanine (i.e. a mononucleotide that has lost its phosphate and ribose components) are not identified, as in previous literature(1), as they have a mass below the minimum specified mass shift specified in the open search parameters used here (150 Da).

Supplementary Table 3: Input data for DisVis simulations. Provided as a separate table in Microsoft Excel xlsx format.

## Supplementary Results

### Optimisation of sample preparation and data analysis

#### Changes to C18 clean-up procedure

We selected the Polypyrimidine Tract Binding Protein 1 (PTBP1) in complex with the internal ribosome entry site (IRES) of the encephalomyocarditis virus (EMCV) as a model for sample preparation, because the protein contains multiple RRM domains, and the RNA is relatively long (88 nucleotides). Together, this results in a large number of amino acid cross-linking sites on the protein, comprising a variety of RNA species linked to them, as previously reported(3). The complex therefore provides an adequate complexity for generalised sample preparation optimisation measures which may be transferred to other complexes. The previously published CLIR-MS sample preparation and analysis procedure(3) was used as a baseline for protocol optimisations.

We first tested the impact of miniaturising the C_18_ solid-phase extraction (SPE) step (**Figure 1a** step 3) on the number of XLSMs made from a PTBP1-IRES CLIR-MS sample. Given the low expected sample amount after MOAC enrichment, we prepared the PTBP1-IRES CLIR-MS sample with regular cartridges as described previously(3), and additionally with the same protocol but with SPE cartridges replaced with Stage tips(4). We then analysed both samples with LC-MS/MS using identical acquisition parameters and compared the number of XLSMs produced by an xQuest search(4) (**Supplementary Figure 1a**). When using Stage tips, the number of XLSMs identified from each replicate increased by approximately 33% (from 129 to 172, mean of both replicates) compared with larger SPE cartridges. This suggests that miniaturising the SPE step, and thus minimising the surface area of vessels in contact with samples, improves recovery of peptide-RNA adducts prior to LC-MS/MS analysis.

#### Changes to mass spectrometry data acquisition

Next, we investigated whether changes to MS acquisition parameters could increase the number of XLSMs provided by a CLIR-MS experiment (**Figure 1a** step 4). Multiple activation methods for MS/MS peptide sequencing are available on modern instruments, some of which may provide superior fragmentation, or preservation of post-translational modifications (PTMs)(5). Assuming that a peptide cross-linked to a short piece of RNA (one to four nucleotides) behaves like a peptide with a PTM during MS analysis, the choice of fragmentation method may therefore influence the number of identified XLSMs. To test this, the aliquots of the same PTBP1-IRES sample were injected multiple times and analysed with three different activation methods, (ion trap) collision induced dissociation (CID), electron transfer/higher-energy collision-induced dissociation (EThcD) and higher-energy collision-induced dissociation (HCD). The numbers of XLSMs returned in each case were compared (**Supplementary Figure 1b**). Injections measured with the EThcD acquisition method produced an average of just 32 identifications per injection (mean of both replicates), representing a decrease on the CID method used previously for CLIR-MS. HCD appeared to perform best, with an average of 118 identifications per MS run (mean of both injections), representing an increase of 74% compared with CID (mean of 68 identifications per injection). Importantly, HCD also appeared better suited to detection of longer RNA adducts, returning a greater number of di- and trinucleotide containing XLSMs than CID or EThcD (**Supplementary** **Figure 1b**). These longer RNA adducts are essential in assigning RNA localisation, given the small number of building blocks (four nucleobases) and the fact that the short oligonucleotide is not directly sequenced in our MS method. Based on these observations, we selected HCD as the default fragmentation method for LC-MS/MS analysis for CLIR-MS.

Subsequently, we also tested LC-MS/MS analysis with varying HCD collision energies (see Methods for details). When measuring performance only by the number of XLSMs identified by the search pipeline, increased energy led to an increase in XLSMs (**Supplementary** **Figure 2a**). However, more careful analysis of the trends revealed that XLSMs at higher energies tended to have a lower proportion of the fragment ion intensity (total ion current, TIC) assigned to expected fragment ion types (**Supplementary** **Figure 2b**). This could be due to increased fragmentation of the nucleotide component of the cross-linked species. Given that we aimed to assign as much of the total signal intensity as possible to make a confident XLSM, we selected a low collision energy of 23% for further experiments.

### Evaluating the impact of perturbations during the UV cross-linking reaction on detected protein-RNA cross-links

We first tested the impact of irradiation energy dose on the outcome of a CLIR-MS experiment. Aliquots of FOX1-FBE sample were subjected to four different doses of 254 nm irradiation energy - 800 mJ/cm^2^, 4000 mJ/cm^2^, 8000 mJ/cm^2^ or 16000 mJ/cm^2^. Cross-linked samples were then prepared and analysed using the optimised CLIR-MS workflow. The identified cross-links were then compared between the irradiation conditions (**Supplementary Figure 3a**). Both major protein sites of cross-linking (clustered around positions 126 and 160) remained consistent, as did the composition of RNA detected at each site, between the conditions. These data suggest that the results obtained from CLIR-MS are qualitatively robust over a broad range of UV irradiation doses.

Next, we used the FOX1-FBE complex to investigate the impact of sample cooling during irradiation on cross-links identified in a CLIR-MS experiment. Four aliquots of complex were irradiated with the same energy at 254 nm (3200 mJ/cm^2^), but each sample was cooled to a different temperature during irradiation, with one cross-linked at room temperature (RT, approximately 25°C), one on ice (approximately 0°C), and one each on metal plates pre-cooled to -20°C and -80°C respectively. After preparation and analysis of the samples by CLIR-MS, we compared the cross-linked protein positions between the temperature conditions (**Supplementary Figure 3b**). As with variation of irradiation dose, the unique identifications (combinations of RNA species and amino acid positions they are linked to) remained qualitatively constant between the conditions, further supporting the robustness of cross-links identified in a CLIR-MS experiment over a broad range of UV cross-linking conditions. Whilst the unique information remained constant, the numbers of XLSMs at each site exhibited some temperature-dependent trends, however these require further investigation with a quantitative pipeline to draw firm conclusions.

### Increasing the number of XLSMs with a deeper understanding of possible UV-XL products

To establish a comprehensive set of detectable RNA-derived peptide modifications, the FOX1-FBE complex was selected. With only a single RRM, it yields a less complex sample with a smaller number of peptides cross-linked to RNA which may improve detection of low abundant RNA-derived products during mass spectrometric analysis. Two FOX1-FBE samples were prepared using the CLIR-MS protocol; one was subjected to 254 nm irradiation to induce cross-links, and the other was left unirradiated as a control. Both samples were analysed by LC-MS/MS, and the data subjected to an “open” modification search using MSFragger(6), to discover all possible mass additions. The detected peptide modifications were grouped into 0.1 Da mass bins, and the number of identifications within each mass bin compared between irradiated and non-irradiated samples (**Supplementary Figure 4a and 4b**) and annotated according to previous literature (**Table 1** and **Supplementary Table 2**). Many RNA-derived modifications were observed in the UV irradiated condition, forming clusters corresponding to expected masses of mono-, di-, tri-, and tetranucleotide RNA species cross-linked to peptides (grey shading, **Supplementary Figure 4a**). As expected, many of the most common peptide mass additions in the irradiated sample were present in both light and heavy isotopic forms, with a delta mass corresponding to that expected from the respective RNA attachment. Some abundant modifications that are not specific to the crosslinking reaction were present in both samples such as +258 Da, a modification commonly observed as a result of His-tagged *in vitro* expressed protein preparations, as used for this protein(7).

The putative list of loss products was then used to define a “closed” xQuest search (for the UV cross-linked samples only), expanding on the set of products used in the original protocol(3). In this search approach, both the light and heavy isotope forms of each proposed RNA species must be detected in order to produce an XLSM, thereby validating putative RNA-derived modification types found in the open modification search. The product types that were subjected to confirmation using the xQuest search are shown in **Table 1**. Putative identifications from the open search that could not be validated are described in **Supplementary Table 2**. Based on the confirmatory results of the xQuest search, we routinely incorporated the expanded product list of validated loss products from **Table 1** into the xQuest search parameters for a CLIR-MS experiment, thereby increasing the number of XLSMs obtained from a CLIR-MS data set compared with the original protocol(3). A comparison of results from two xQuest searches of the UV-XL condition data, one using the restricted set of modifications as previously published(3), and one using the expanded set presented here (validated in **Table 1**), is shown in **Supplementary Figure 4c**, and is additionally summarised in **Figure 1c**. The products considered for data analysis can be adjusted in the package RNxQuest(8) to find optimum between recovered identifications and search space expansion.

## Supplementary Figures


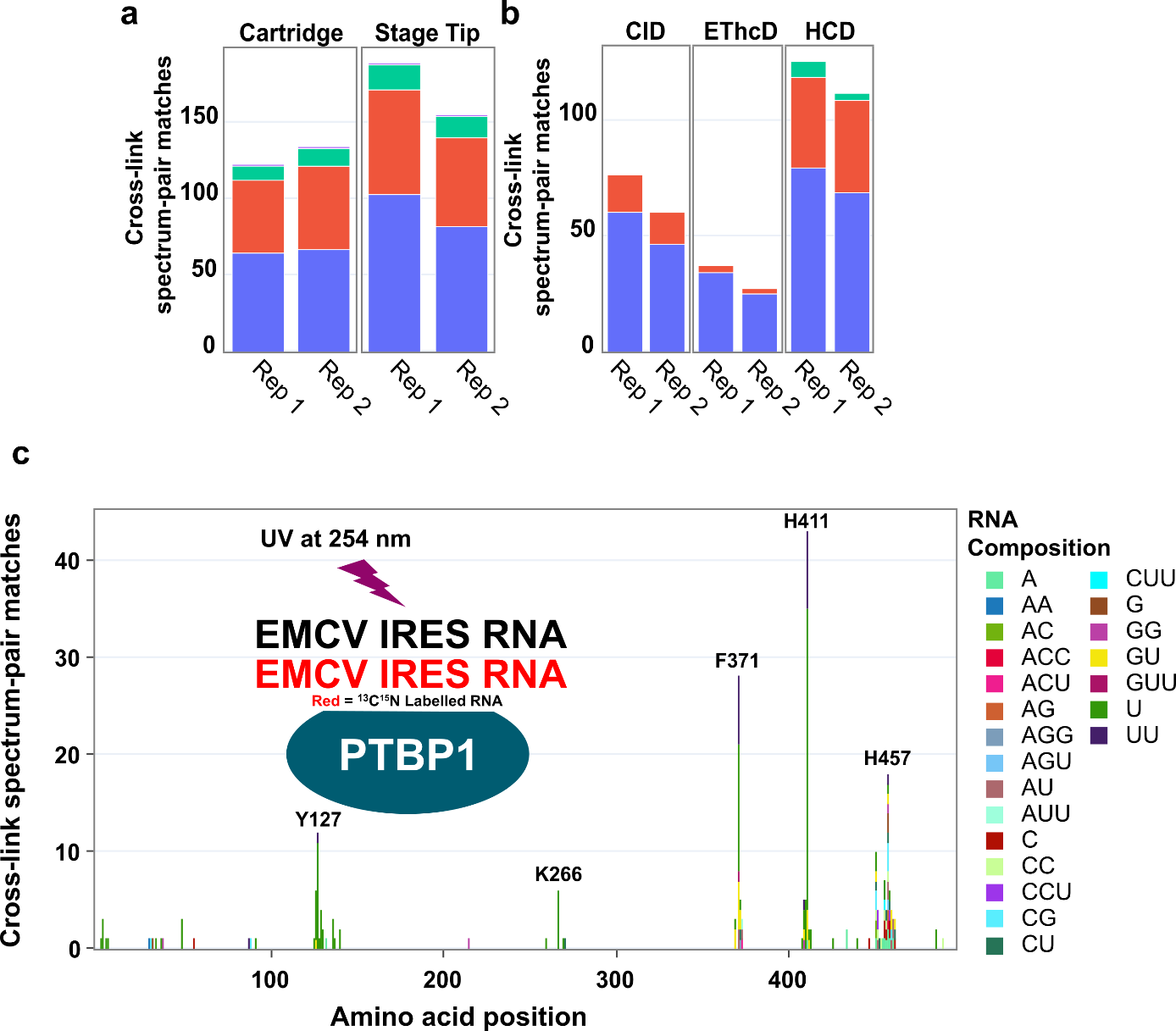


Supplementary Figure 1: Relative contributions of changes to C18 clean-up and MS data acquisition to increased XL numbers.

a) Comparison of the number of XLSMs made from PTBP1-IRES samples prepared using (conventional) cartridges and Stage tips for the final C_18_ clean-up step. Rep = Replicate

b) Comparison of analysis of a single PTBP1-IRES CLIR-MS sample utilising different activation types for peptide fragmentation. Rep = Replicate.

c) Structural information obtained from the PTBP1-IRES complex using the optimal experimental conditions in panels a) and b) respectively. Overlaid, schematic representations of the PTBP1 protein and EMCV IRES RNA in complex, used for data in panels a), b), and c).

a b


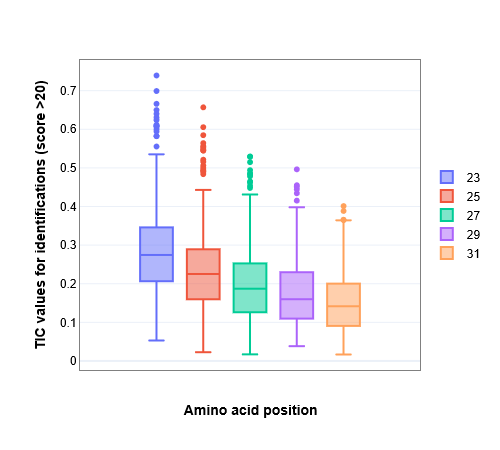

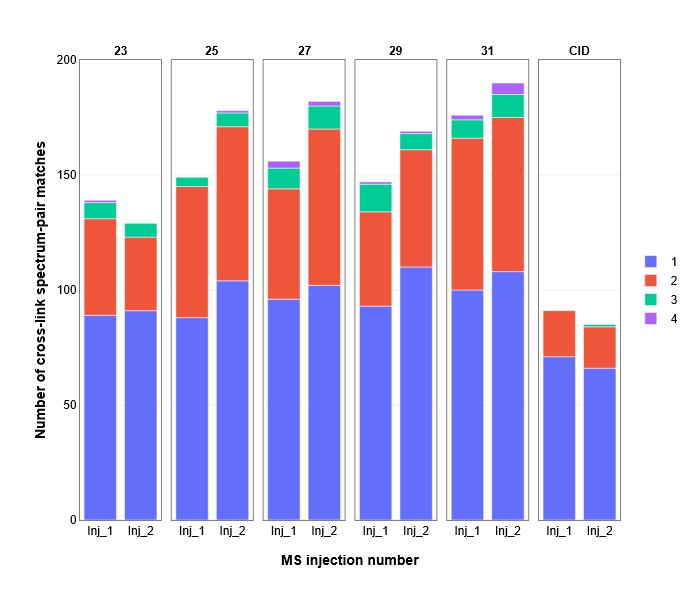


Supplementary Figure 2: Optimisation of HCD collision energy (range, 23-31% as indicated) for mass spectrometry analysis

a) Comparisons of identifications made per MS run of the same PTBP1-IRES CLIR-MS sample, but with different HCD activation energies. Different lengths of nucleotide adducts (1-4) are indicated by different colours according to the legend on the right. Inj = LC-MS injection.

b) Distributions of TIC values for identifications made in xQuest for each HCD activation energy in panel a.


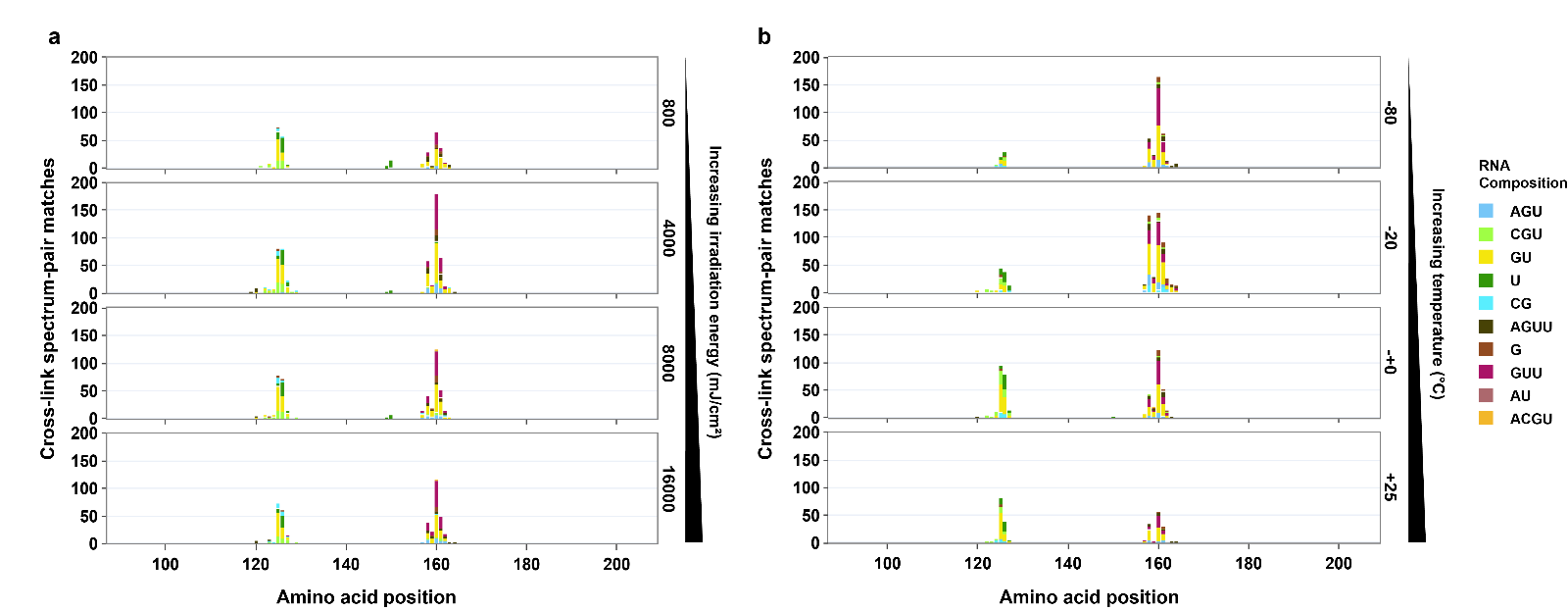


Supplementary Figure 3: The impact of variation in experimental parameters on CLIR-MS results

a)-b) Comparison of results from CLIR-MS analysis of FOX1-FBE complex with a) different UV irradiation energies and b) different cross-linking reaction temperatures.

Whilst the unique identifications (i.e. unique combinations of amino acid positions and RNA compositions) remain mostly constant with changing sample temperature, the numbers of XLSMs at each of the major protein cross-linking sites appear to change depending on temperature. As the temperature increases, the number of identifications at position F126 seems to increase, from 19 in the samples irradiated at -80 °C to 82 in the samples irradiated at room temperature (sum of two replicates). Conversely, the number of identifications around F160 tends to decrease, from 163 at -80 °C to just 57 at room temperature. The trends are also reflected in the amino acid positions immediately surrounding these specific residues and may reveal the suitability of UV cross-linking-based workflows to study dynamics of protein-RNA interactions.

**
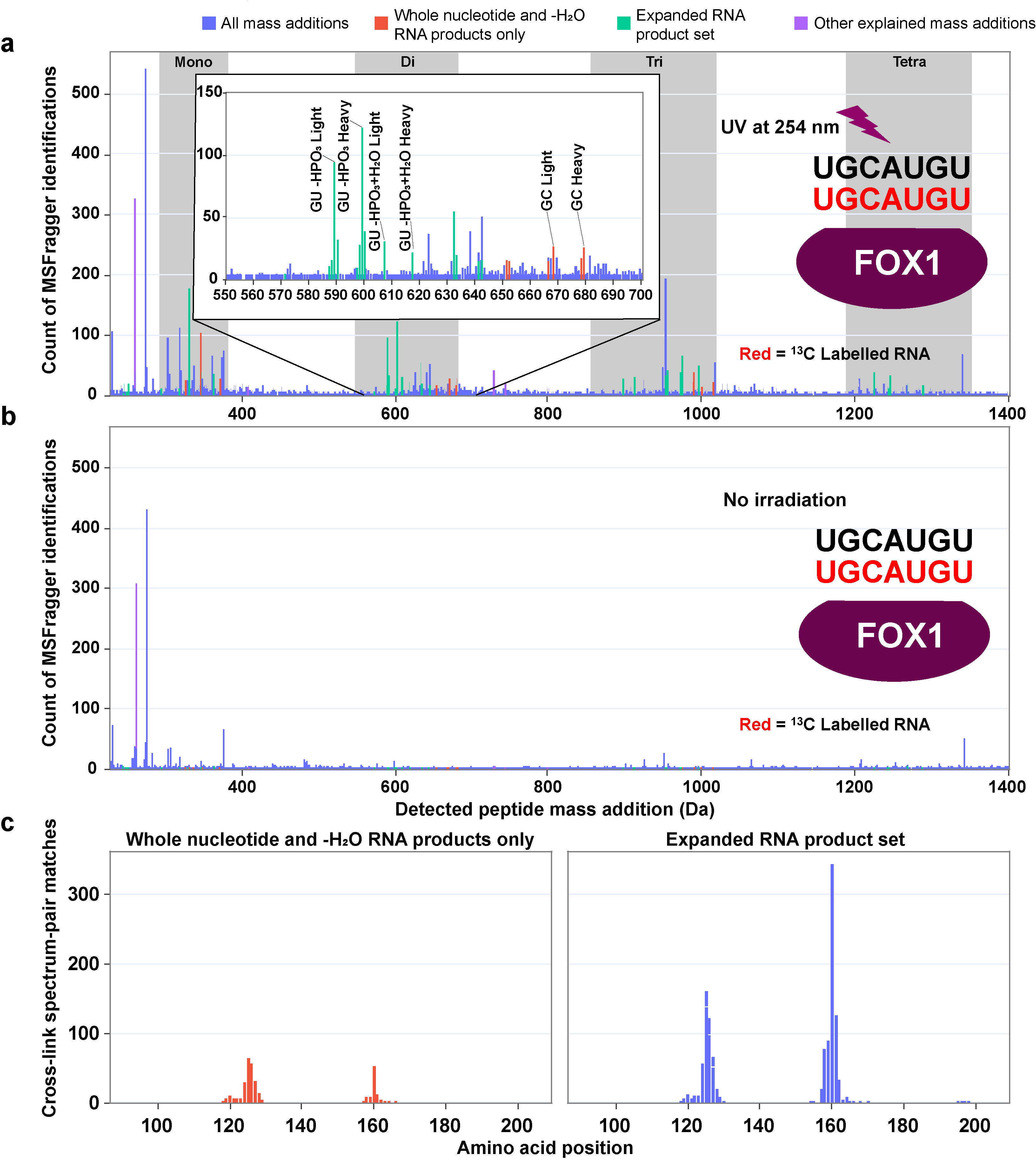
**

Supplementary Figure 4: Optimisation of data analysis through better understanding of the cross-linking products.

a) and b) Open modification searches of the data produced from the a) irradiated and b) unirradiated complexes (data from FOX1-FBE). Any peptide mass additions between 150 and 1400 Da were considered by the search software. The number of identifications found corresponding to each 0.1 Da mass bin is shown. Regions corresponding to mono- (~300-400 Da), di- (~600-700 Da), tri- (~900-1000 Da) and tetranucleotide (~1200-1300 Da) adducts are highlighted in grey. The bars for each mass bin are coloured according to whether they have been used in the original CLIR-MS study(3) or in the present work.

c) Comparison of closed xQuest searches of the same FOX1-FBE data from a) using the entire set of cross-linking products identified in this work (right) in comparison to the modifications specified previously (left)(3).


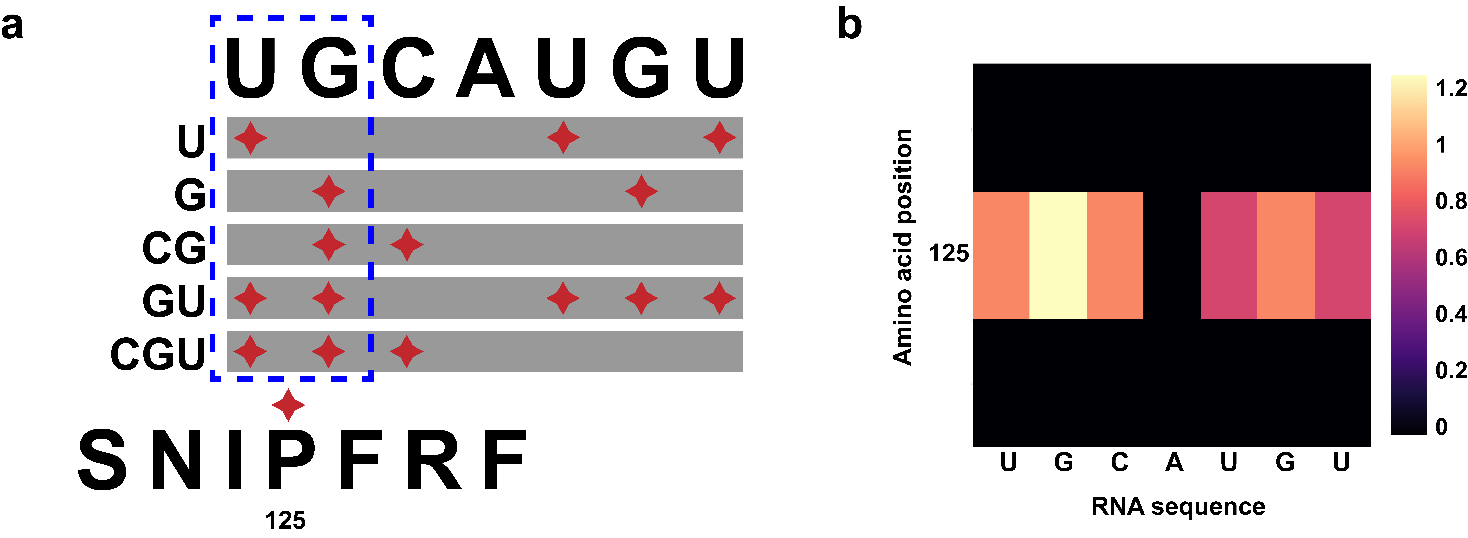


Supplementary Figure 5: Illustration of inference of RNA cross-link position from overlapping polynucleotide adducts, using FOX1-FBE as an example.

a) All RNA compositions detected at amino acid position P125 of the FOX1 RRM are shown, aligned with the full labelled RNA sequence. The combined information from mono-, di- and trinucleotide adducts detected at this amino acid site means the nucleotide positions that form cross-links must be positions uracil 1 and guanine 2 in the RNA sequence.

b) A heatmap representation of the data from the single amino acid position and detected cross-linked nucleotide compositions shown in (a). This concept is systematically extended to every detected cross-linked amino acid position selected in SF3-UBL, in Supplementary Figure 7.


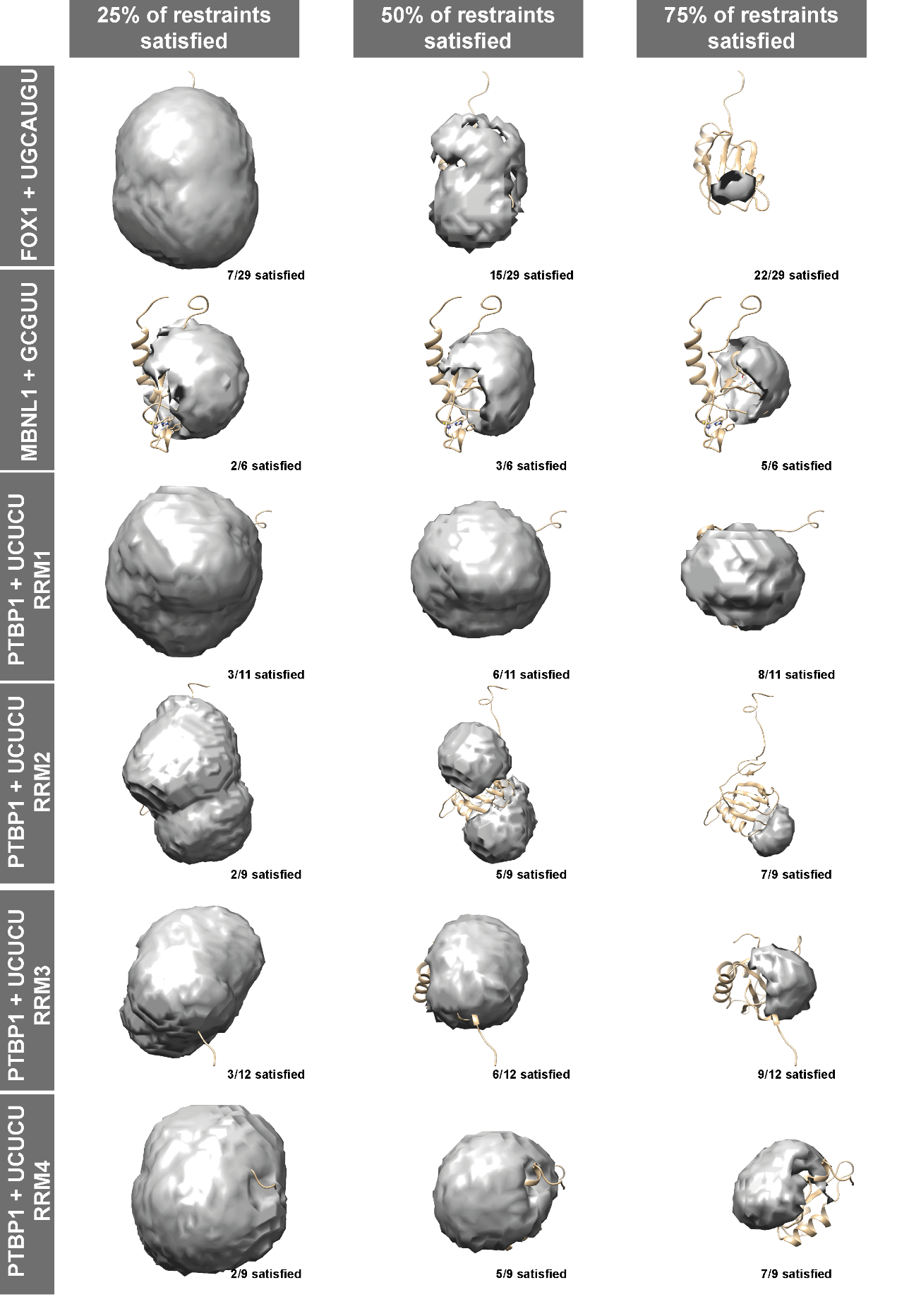


Supplementary Figure 6: Inclusion of larger numbers of restraints leads to a more restricted occupancy space for RNA relative to a protein.

Intuitively, including a greater number of restraints more severely restricts the permitted RNA positions. Here, permitted DisVis occupancy spaces are shown for the canonical RNA binding proteins - FOX1, MBNL1 and PTBP1 model complexes - where arbitrary thresholds of 25%, 50% and 75% of satisfied input restraints are used to define the space. Visualisations produced using UCSF Chimera.


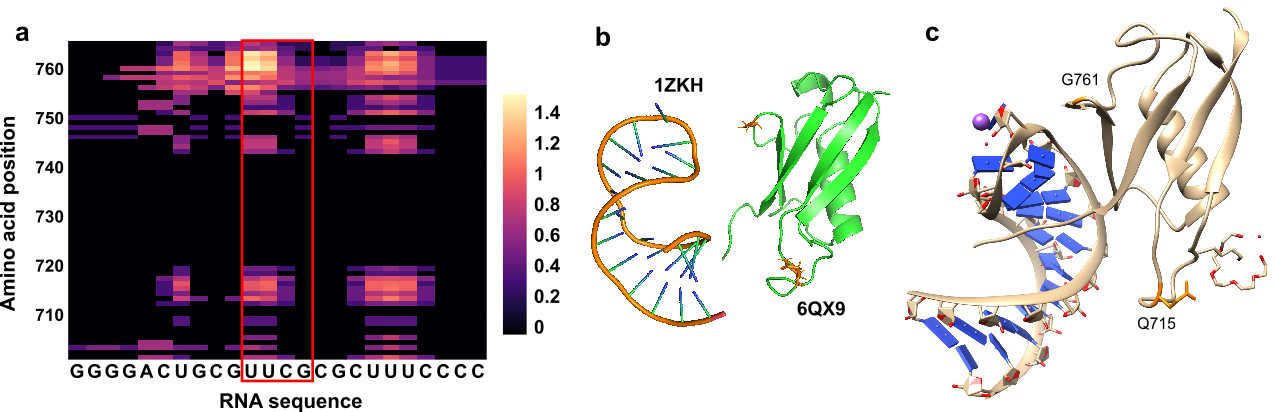


Supplementary Figure 7: Further information relating to CLIR-MS derived XLs describe a non-canonical protein-RNA interaction in SF3A1-UBL.

a) Non-redundant amino acid position and RNA composition XL combinations, systematically overlaid for every XLSM (as described in Supplementary Figure 5) to describe possible RNA interaction sites suggested by CLIR-MS data.

b) Previously published structural models of unbound SF3A1-UBL (PDB ID: 1ZKH) and the U1 snRNA SL4 (PDB ID: 6QX9). Highly cross-linked amino acids from Figure 2k are colored orange. Structures visualized with PyMOL.

c) Structure of the SF3A1-UBL interacting with the U1 snRNA SL4, determined using X-ray crystallography (described separately(9)). Visualization produced using UCSF Chimera.

### LC-MS analysis of chemically synthesised RNA

| **Spectrum number** | **Sequence** | **Mass calculated** | **Mass found** | **Purity** |
| --- | --- | --- | --- | --- |
| 1 | UGCAUGU | 2181.4 | 2180.89 | >99% |
| 2 | *U*G*C*A*U*G*U | 2216.4 | 2215.4 | >99% |
| 3 | ^4S^UGCAUGU | 2197.4 | 2196.38 | >95% |
| 4 | UGCA^4S^UGU | 2197.4 | 2196.84 | >97% |
| 5 | UGCAUG^4S^U | 2197.4 | 2196.84 | >98% |
| 6 | ^4S^U*G*C*A*U*G*U | 2227.4 | 2226.37 | >97% |
| 7 | *U*G*C*A^4S^U*G*U | 2227.4 | 2226.37 | >99% |
| 8 | *U*G*C*A*U*G^4S^U | 2227.4 | 2226.37 | >98% |
| 9 | CGCUU | 1505.9 | 1505.52 | >99% |
| 10 | *C*G*C*U*U | 1530.9 | 1530.19 | >99% |
| 11 | UCUCU | 1466.9 | 1465.3 | >99% |
| 12 | *U*C*U*C*U | 1491.9 | 1490.2 | >99% |

1. UGCAUGU


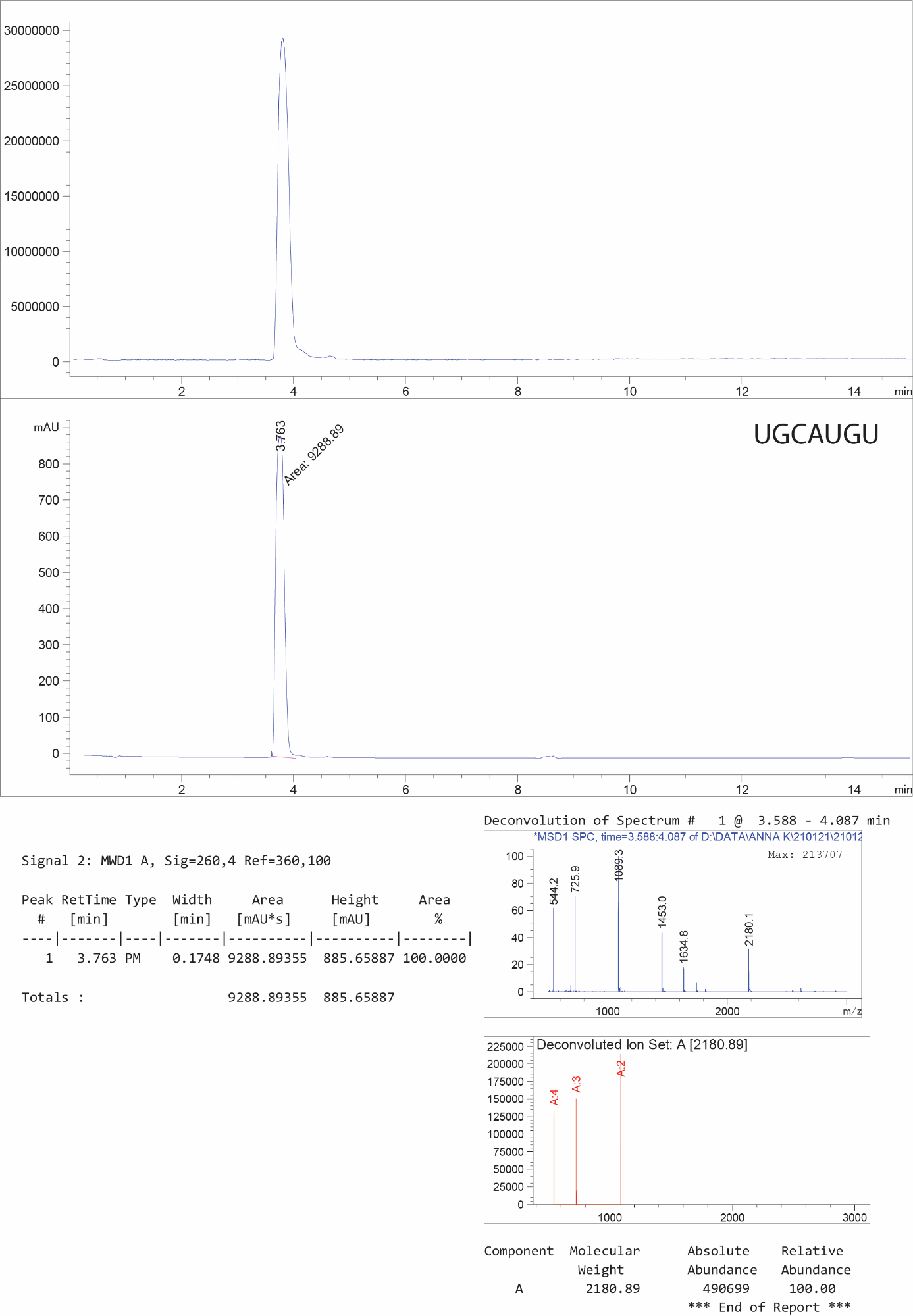


1. ^13C^U^13C^G^13C^C^13C^A^13C^U^13C^G^13C^U


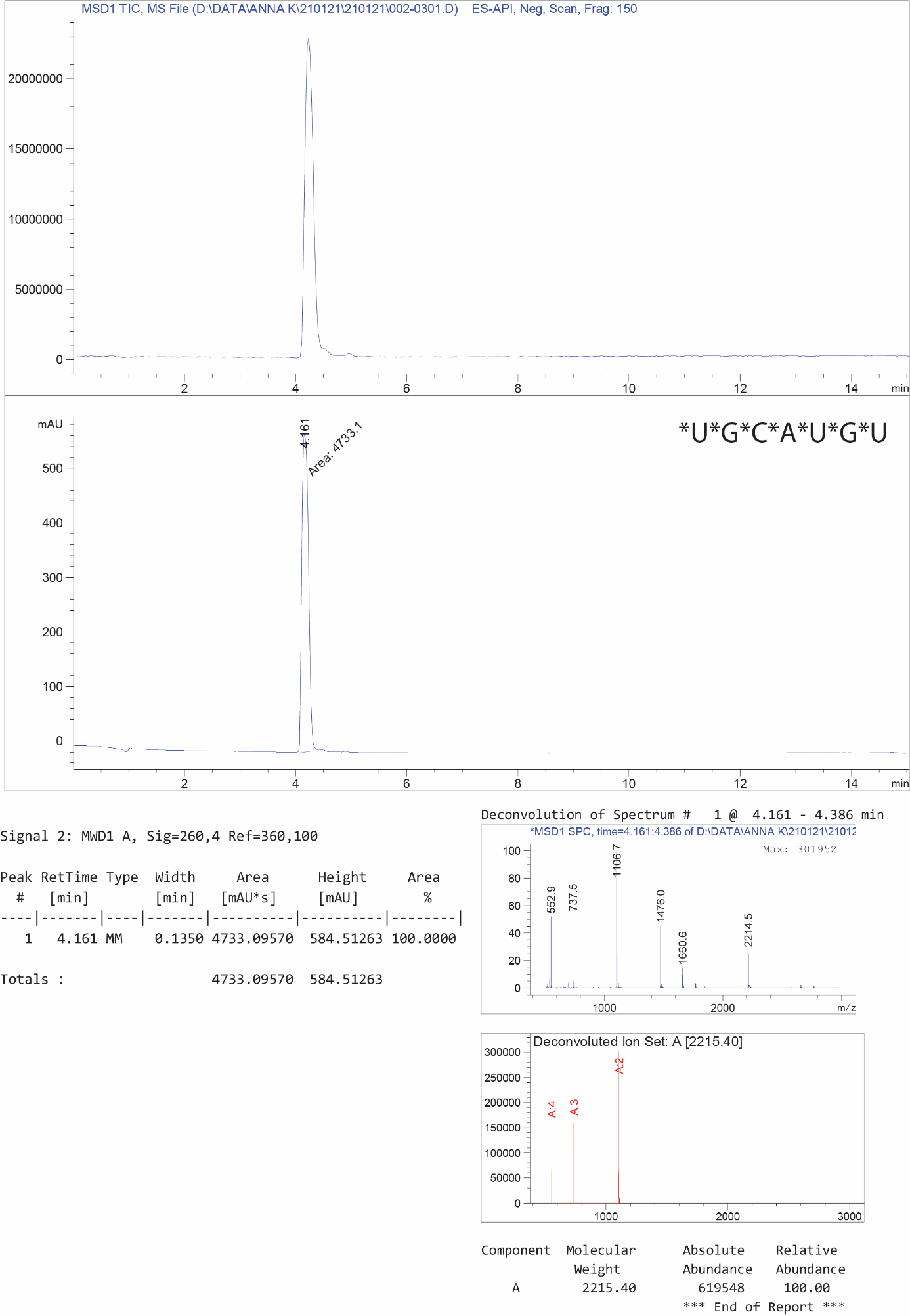


1. ^4S^UGCAUGU


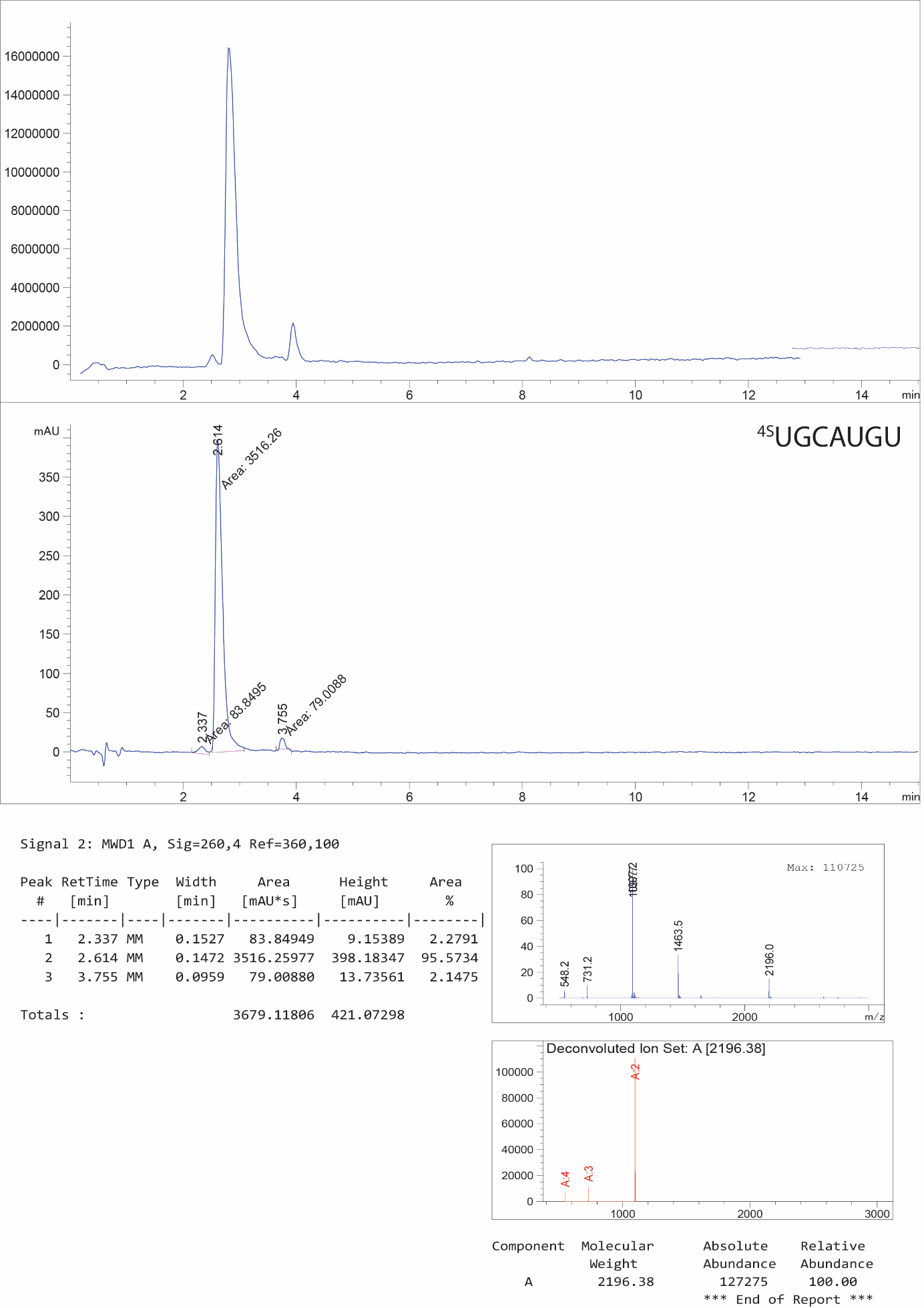


1. UGCA^4S^UGU


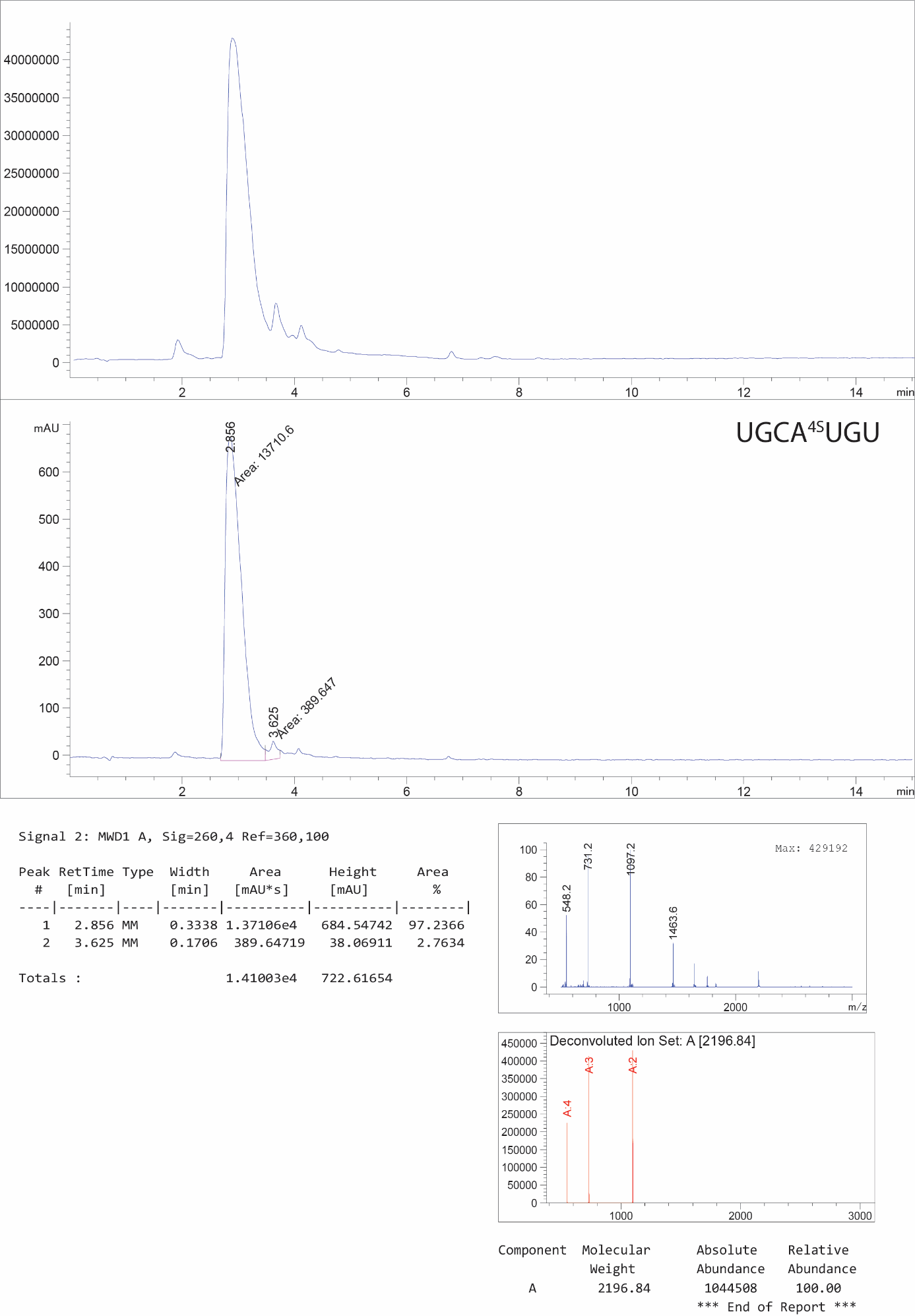


1. UGCAUG^4S^U


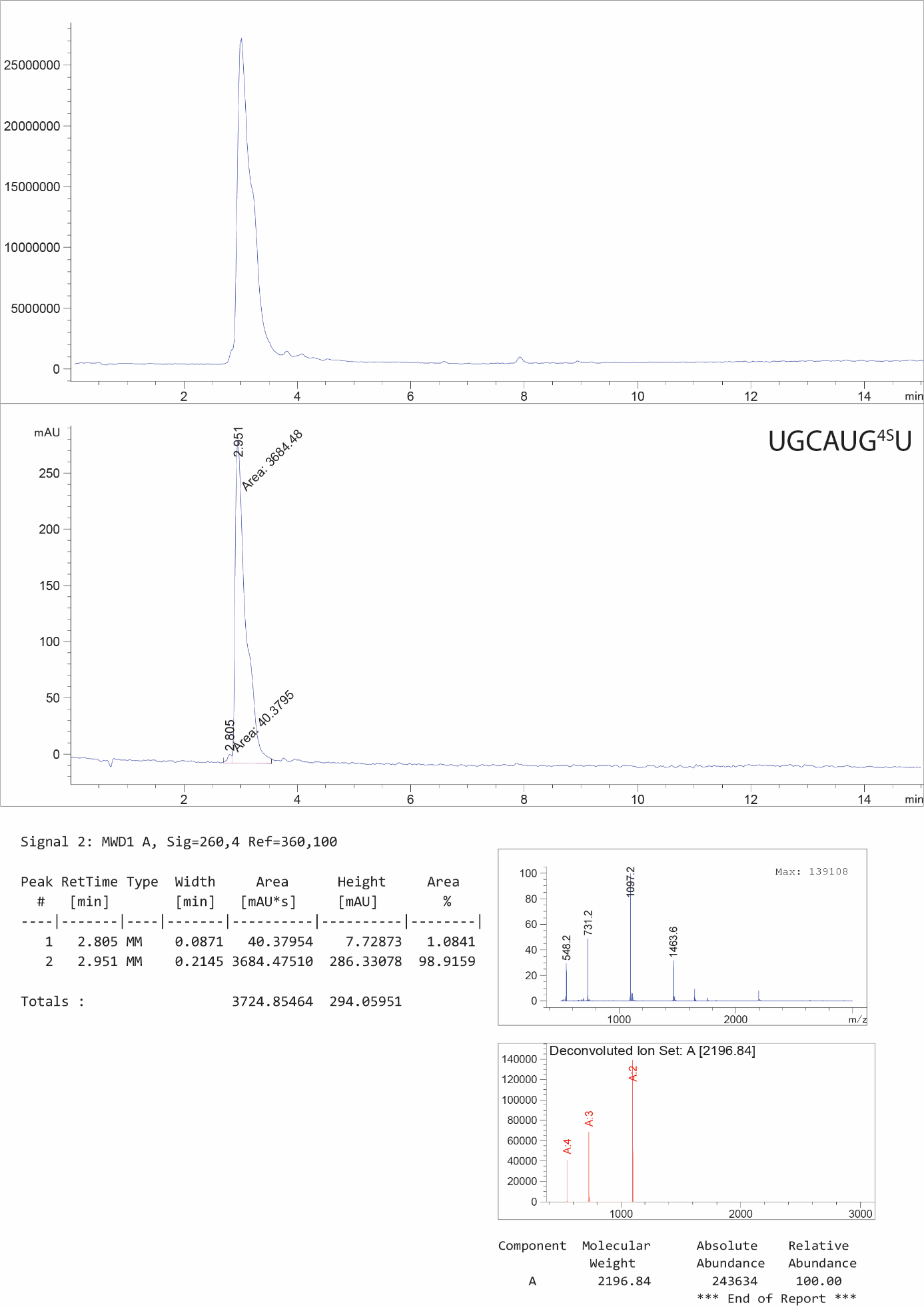


1. ^4S^U^13C^G^13C^C^13C^A^13C^U^13C^G^13C^C^13C^U


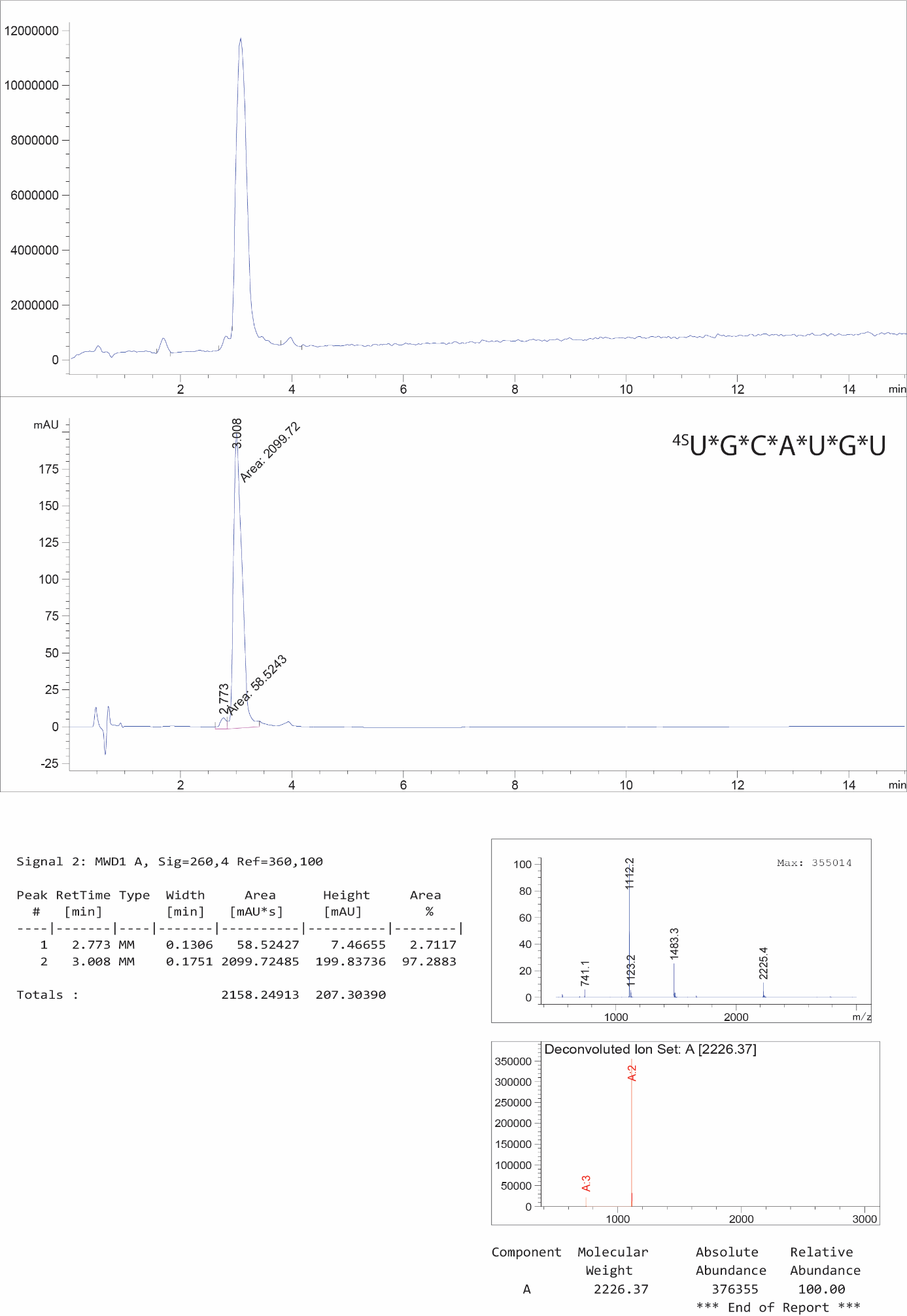


1. ^13C^U^13C^G^13C^C^13C^A^4S^U^13C^G^13C^ U


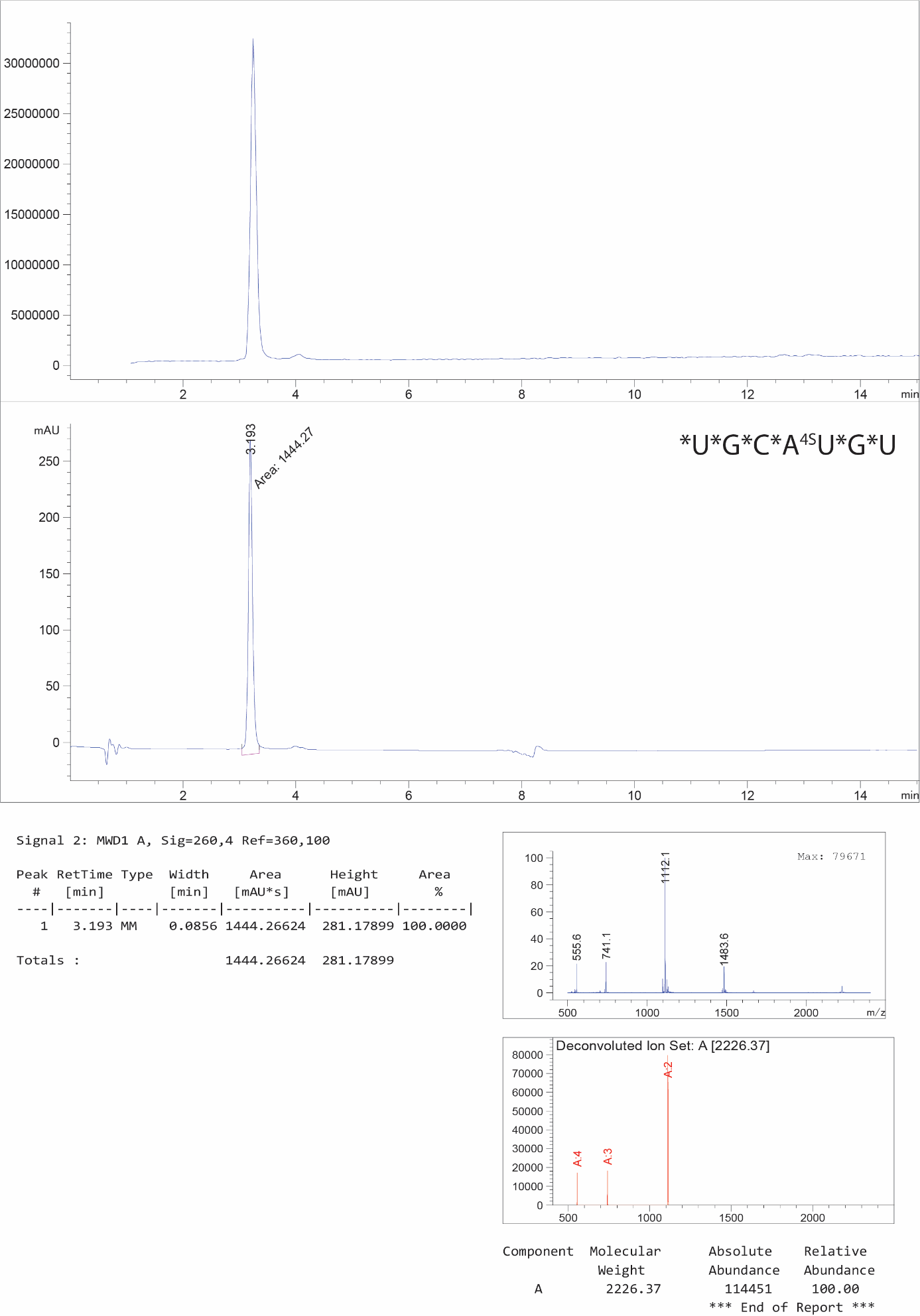


1. ^13C^U^13C^G^13C^C^13C^A^13C^U^13C^G^4S^U


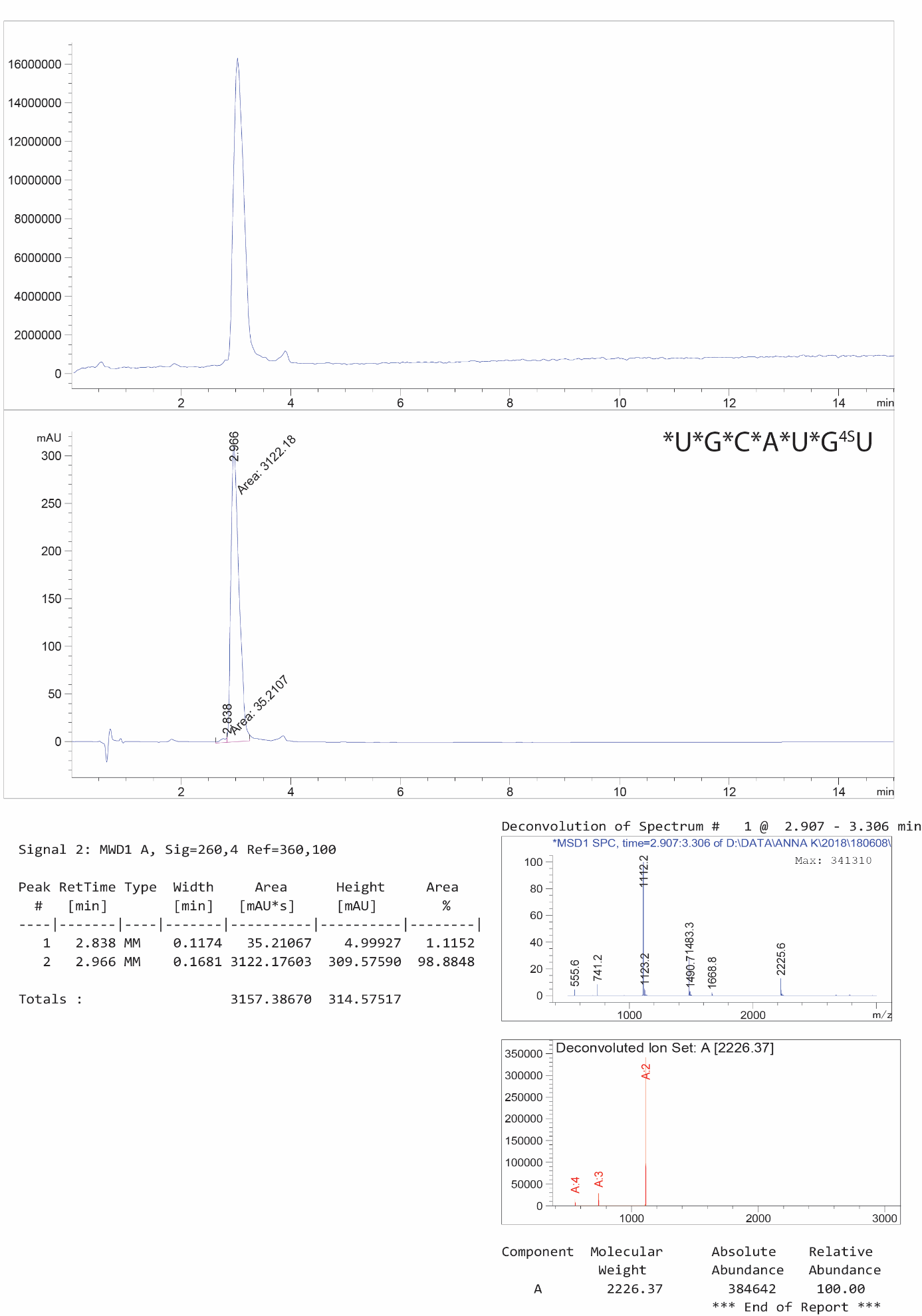


1. CGCUU


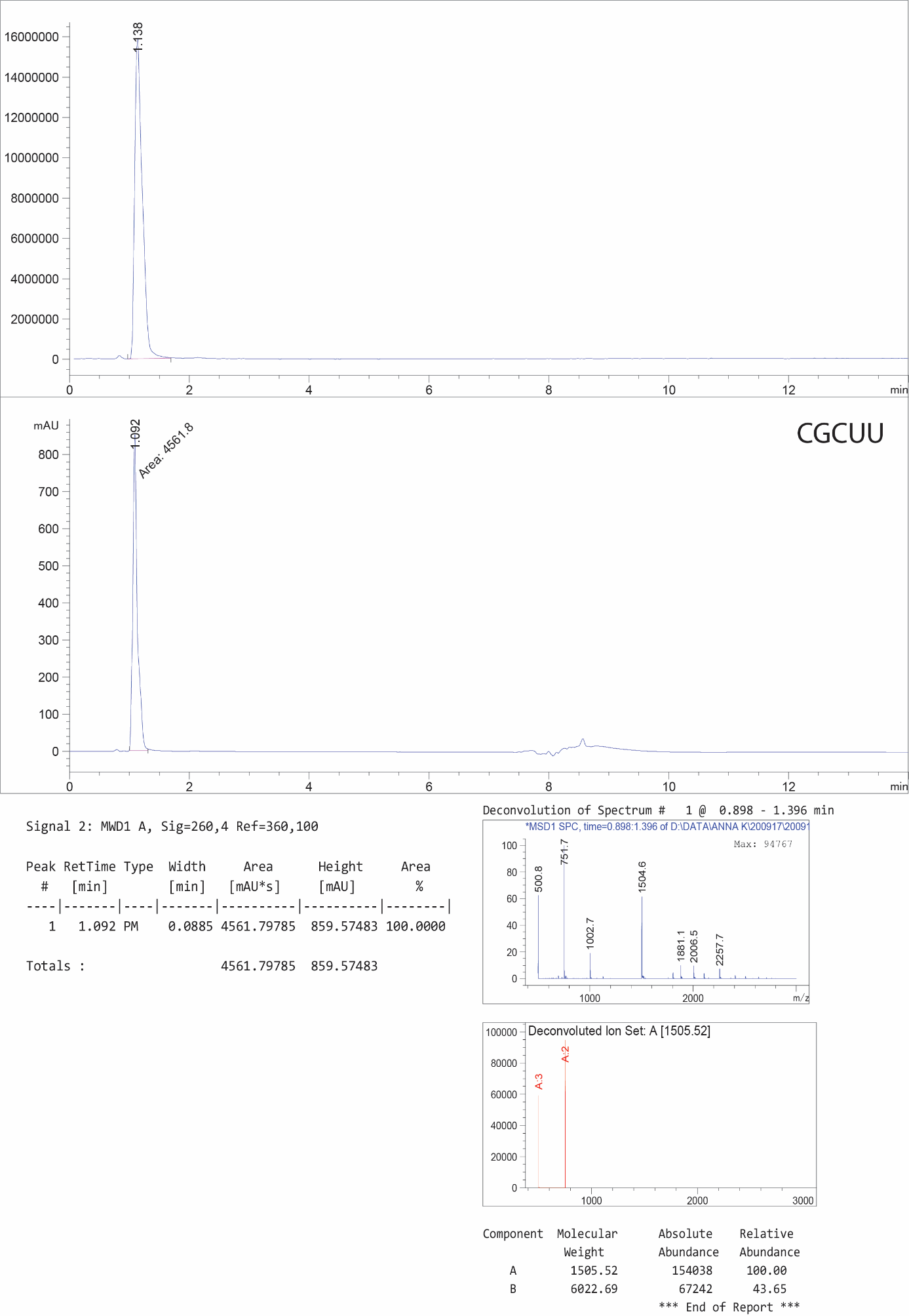


1. ^13C^C^13C^G^13C^C^13C^U^13C^U


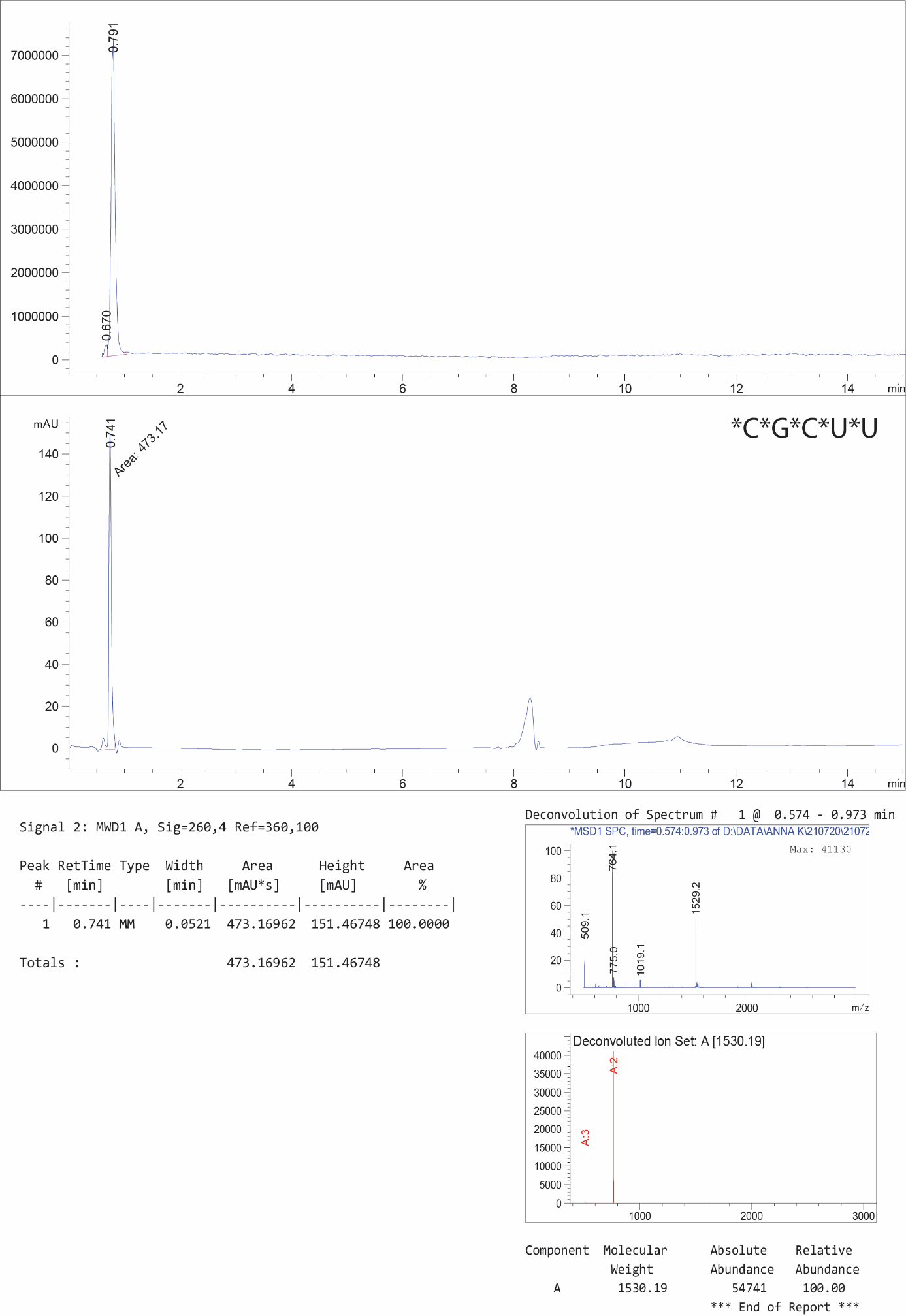


1. UCUCU


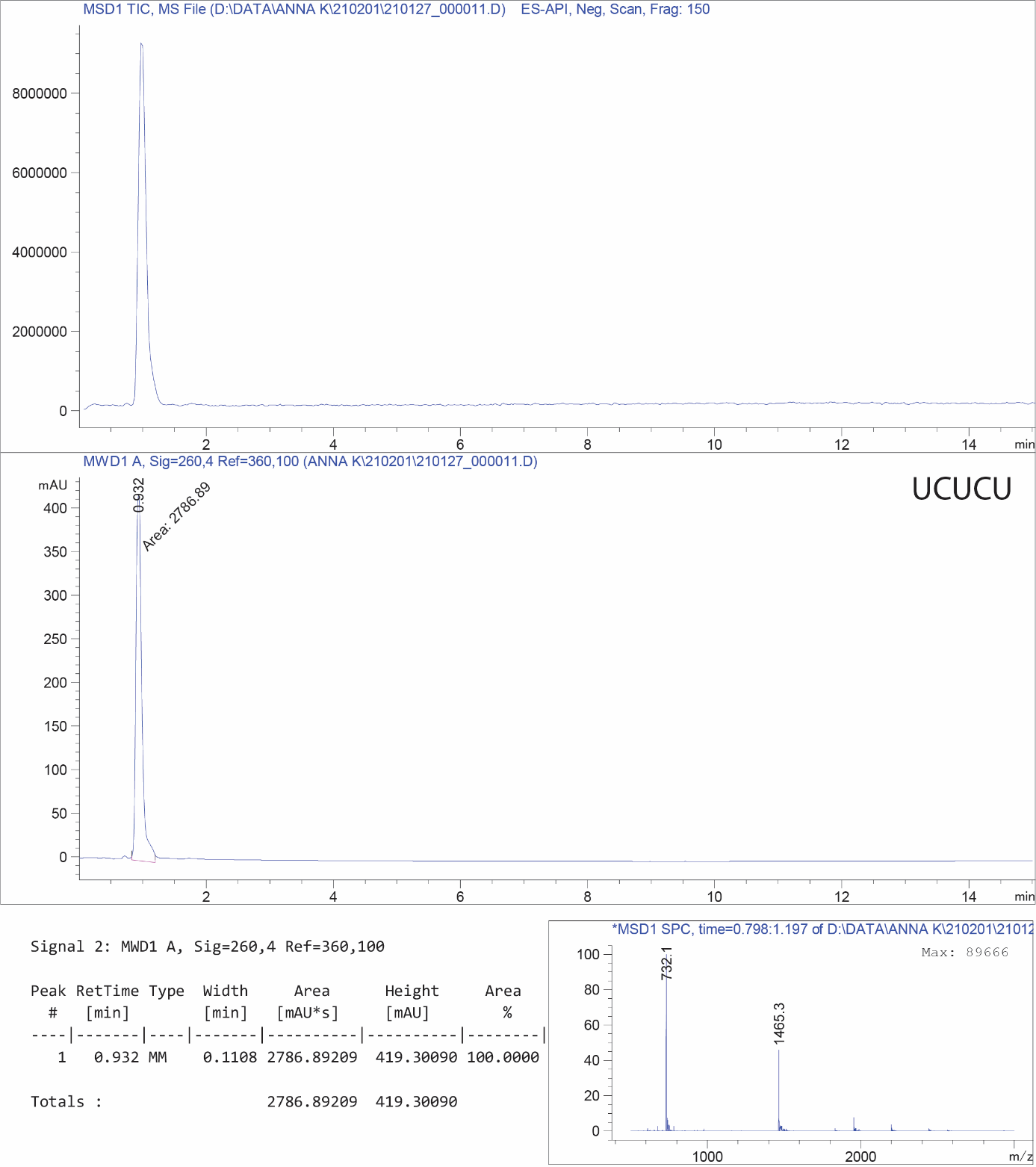


1. ^13C^U^13C^C^13C^U^13C^C^13C^U


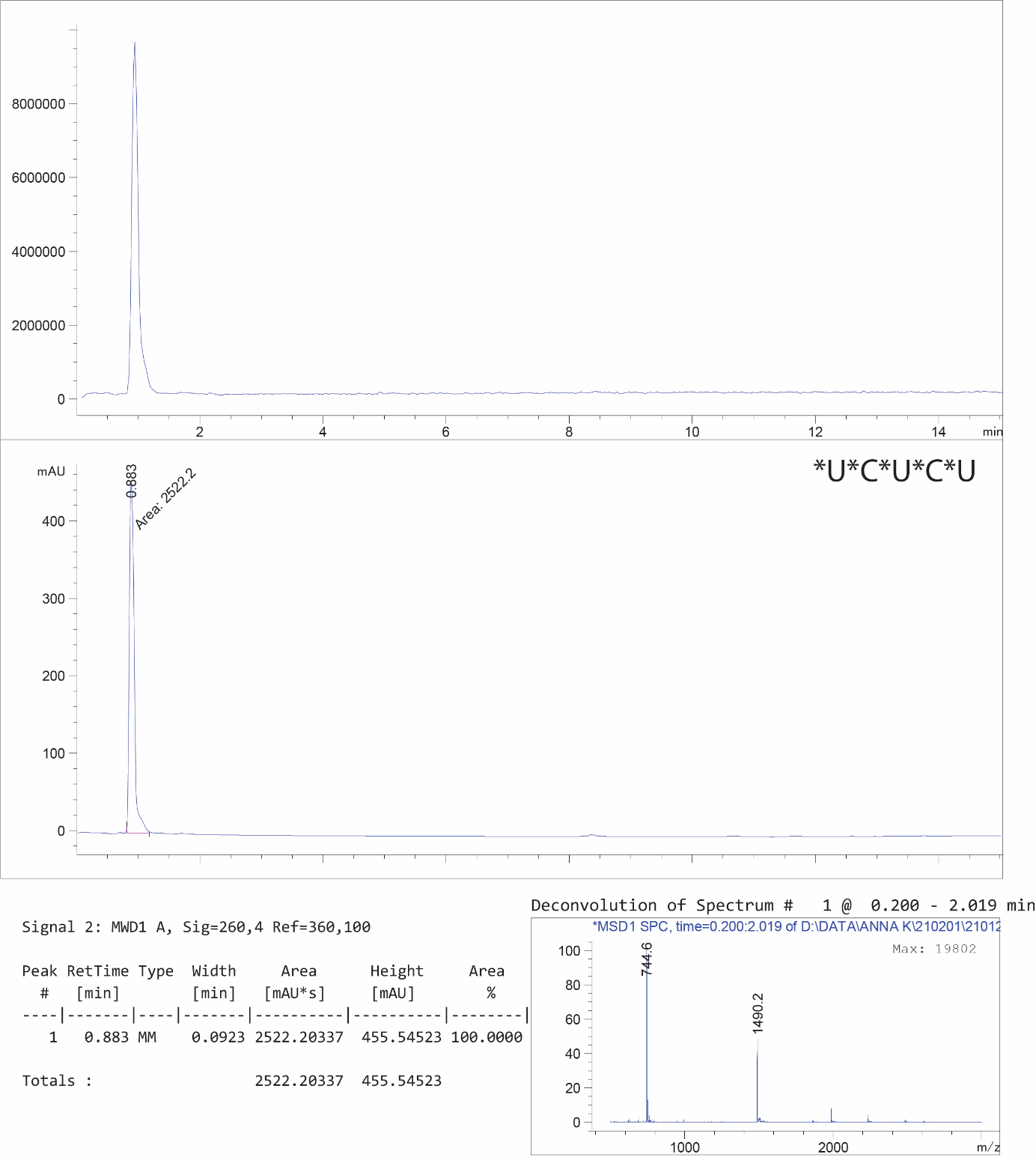


## Supplementary References

1. Panhale,A., Richter,F.M., Ramírez,F., Shvedunova,M., Manke,T., Mittler,G. and Akhtar,A. (2019) CAPRI enables comparison of evolutionarily conserved RNA interacting regions. *Nat. Commun.*, **10**, 2682.

2. Kramer,K., Hummel,P., Hsiao,H.H., Luo,X., Wahl,M. and Urlaub,H. (2011) Mass-spectrometric analysis of proteins cross-linked to 4-thio-uracil- and 5-bromo-uracil-substituted RNA. *Int. J. Mass Spectrom.*, **304**, 184–194.

3. Dorn,G., Leitner,A., Boudet,J., Campagne,S., von Schroetter,C., Moursy,A., Aebersold,R. and Allain,F.H.-T.H.T.H.-T. (2017) Structural modeling of protein-RNA complexes using crosslinking of segmentally isotope-labeled RNA and MS/MS. *Nat. Methods*, **14**, 487–490.

4. Rinner,O., Seebacher,J., Walzthoeni,T., Mueller,L.N., Beck,M., Schmidt,A., Mueller,M. and Aebersold,R. (2008) Identification of cross-linked peptides from large sequence databases. *Nat. Methods*, **5**, 315–318.

5. Frese,C.K., Altelaar,A.F.M., Hennrich,M.L., Nolting,D., Zeller,M., Griep-Raming,J., Heck,A.J.R. and Mohammed,S. (2011) Improved Peptide Identification by Targeted Fragmentation Using CID, HCD and ETD on an LTQ-Orbitrap Velos. *J. Proteome Res.*, **10**, 2377–2388.

6. Kong,A.T., Leprevost,F. V., Avtonomov,D.M., Mellacheruvu,D. and Nesvizhskii,A.I. (2017) MSFragger: Ultrafast and comprehensive peptide identification in mass spectrometry-based proteomics. *Nat. Methods*, **14**, 513–520.

7. Geoghegan,K.F., Dixon,H.B.F., Rosner,P.J., Hoth,L.R., Lanzetti,A.J., Borzilleri,K.A., Marr,E.S., Pezzullo,L.H., Martin,L.B., Lemotte,P.K., *et al.* (1999) Spontaneous α-N-6-phosphogluconoylation of a ‘His tag’ in Escherichia coli: The cause of extra mass of 258 or 178 Da in fusion proteins. *Anal. Biochem.*, **267**, 169–184.

8. Sarnowski,C.P., Götze,M. and Leitner,A. (2023) RNxQuest : An Extension to the xQuest Pipeline Enabling Analysis of Protein–RNA Cross-Linking/Mass Spectrometry Data. *J. Proteome Res.*, **22**, 3368-3382.

9. de Vries,T., Martelly,W., Campagne,S., Sabath,K., Sarnowski,C.P., Wong,J., Leitner,A., Jonas,S., Sharma,S. and Allain,F.H.-T. (2022) Sequence-specific RNA recognition by an RGG motif connects U1 and U2 snRNP for spliceosome assembly. *Proc. Natl. Acad. Sci.*, **119**, e2114092119.
